# *Porphyromonas gingivalis* Regulates TREM-1 in Human Polymorphonuclear Neutrophils via Its Gingipains

**DOI:** 10.1371/journal.pone.0075784

**Published:** 2013-10-04

**Authors:** Nagihan Bostanci, Thomas Thurnheer, Joseph Aduse-Opoku, Michael A. Curtis, Annelies S. Zinkernagel, Georgios N. Belibasakis

**Affiliations:** 1 Oral Translational Research, Institute of Oral Biology, Center of Dental Medicine, University of Zürich, Zürich, Switzerland; 2 Oral Microbiology and Immunology, Institute of Oral Biology, Center of Dental Medicine, University of Zürich, Zürich, Switzerland; 3 Centre for Immunology and Infectious Disease, Blizard Institute, Barts and the London School of Medicine and Dentistry, Queen Mary University of London, London, United Kingdom; 4 Division of Infectious Diseases and Hospital Epidemiology, University Hospital Zürich, University of Zürich, Zürich, Switzerland; University of Toronto, Canada

## Abstract

The Triggering Receptor Expressed on Myeloid cells 1 (TREM-1) is a cell surface receptor of the immunoglobulin superfamily, with the capacity to amplify pro-inflammatory cytokine production and regulate apoptosis. Polymorphonuclear neutrophils (PMNs) are the first line of defence against infection, and a major source of TREM-1. *Porphyromonas gingivalis* is a Gram-negative anaerobe highly implicated in the inflammatory processes governing periodontal disease, which is characterized by the destruction of the tooth-supporting tissues. It expresses a number of virulence factors, including the cysteine proteinases (or gingipains). The aim of this *in vitro* study was to investigate the effect of *P. gingivalis* on TREM-1 expression and production by primary human PMNs, and to evaluate the role of its gingipains in this process. After 4 h of challenge, *P. gingivalis* enhanced TREM-1 expression as identified by quantitative real-time PCR. This was followed by an increase in soluble (s)TREM-1 secretion over a period of 18 h, as determined by ELISA. At this time-point, the *P. gingivalis*-challenged PMNs exhibited diminished TREM-1 cell-membrane staining, as identified by flow cytometry and confocal laser scanning microscopy. Furthermore engagement of TREM-1, by means of anti-TREM-1 antibodies, enhanced the capacity of *P. gingivalis* to stimulate interleukin (IL)-8 production. Conversely, antagonism of TREM-1 using a synthetic peptide resulted in reduction of IL-8 secretion. Using isogenic *P. gingivalis* mutant strains, we identified the Arg-gingipain to be responsible for shedding of sTREM-1 from the PMN surface, whereas the Lys-gingipain had the capacity to degrade TREM-1. In conclusion, the differential regulation of TREM-1 by the *P. gingivalis* gingipains may present a novel mechanism by which *P. gingivalis* manipulates the host innate immune response helping to establish chronic periodontal inflammation.

## Introduction

Periodontal diseases are the most common inflammatory infections in humans, caused by complex polymicrobial biofilms attaching on the tooth surface and causing inflammation by the tooth-supporting (periodontal) tissues [1]. The balanced relationship between the biofilm microbiota and the host response of periodontal tissues is commensurate with health. In contrast, a dysbiotic relationship can lead to periodontal disease [2], which is characterized by the destruction of the periodontal tissues (periodontitis), and eventually tooth loss. Polymorphonuclear neutrophils (PMNs) are the first cells to be recruited to the site of inflammation, in response to the developing subgingival biofilm-associated infections in the periodontal tissues [3], [4]. Beyond the protective role against bacterial infections PMN may also cause collateral damage to the periodontal tissues. Although PMNs have been extensively studied for their involvement in the local inflammatory responses to periodontal disease, not much is known on their potential role in the amplification of inflammation.

The Triggering Receptor Expressed on Myeloid cells 1 (TREM-1) is a cell surface receptor of the immunoglobulin superfamily, constitutively expressed by monocytes and PMN [5]. It is activated upon bacterial recognition by host cells, triggering a number of intracellular signalling events that result in enhanced pro-inflammatory cytokine production [5], [6]. Bacterial or fungal infections can cause up-regulation of membrane bound TREM-1, as well as release in its soluble (s)TREM-1 form [7], [8] rendering it a useful early inflammatory biomarker for systemic infection [9]. Recent evidence in periodontitis patients demonstrated elevated sTREM-1 salivary and serum levels [10], or gingival crevicular fluid levels [11], positively associated with the presence of putative periodontal pathogens in subgingival biofilms [12].

The Gram-negative anaerobe bacterium *Porphyromonas gingivalis,* is one of the major pathogens implicated in the chronic inflammatory responses governing periodontal disease by either impeding or modulating innate immune defence mechanisms in the periodontium [13]–[15]. Since *P. gingivalis* shifts the commensal biofilm composition towards a dysbiotic flora resulting in pathological host response and subsequently in periodontitis it is also known as “keystone-pathogen” [16], [17]. Important *P. gingivalis* virulence factors that deregulate innate immune responses are the potent arginine-specific and lysine-specific cysteine proteinases aka “gingipains” [18]–[20].

It was recently shown that *P. gingivalis* induces TREM-1 expression in monocytes, concomitantly with an increased release of sTREM-1 [21], [22]. Also, mice inoculated with *P. gingivalis* exhibited higher TREM-1 gene expression, compared to their corresponding uninfected controls [16]. In PMN TREM-1 activation was shown to propagate degranulation, respiratory burst, phagocytosis, and cytokine release in response to bacterial infections with *Staphylococcus aureus* and *Pseudomonas aeruginosa* [23], [24]. Accordingly, in the periodontal pocket the local excessive inflammatory reaction of the PMNs in response to the “keystone pathogen” *P. gingivalis* could be regulated by TREM-1. The aim of this study was to investigate the effect of *P. gingivalis* on TREM-1 regulation in PMNs and to evaluate the involvement of its gingipains in this process.

## Materials and Methods

### Bacterial Growth Conditions

*Porphyromonas gingivalis* wild-type W50 strain and gingipain knock-out mutant K1A and E8 strains were used in this study [18], [19], [25]. E8 strain is deficient in both Arg-gingipain A and Arg-gingipain B (*rgpArgpB*), whereas K1A strain is deficient in Lys-gingipain (*kgp*). All three strains were grown anaerobically on Columbia Blood Agar (CBA) plates for 3 to 4 days at 37°C, followed by further anaerobic sub-culturing for 2 to 3 days at 37°C in Brain Heart Infusion broth, containing 0.5% hemin and 0.2% menadione.

### Isolation of PMN and Bacterial Challenge

Blood was taken from healthy donors (BD Vacutainer, Allschwil, Switzerland) and PMNs were isolated by density gradient centrifugation using the PolymorphprepTM system according to the manufacturer`s protocol (Axis-Shield) and in strict accordance to the protocol 2010–0126/0, approved by the Institutional Review Board of the University of Zürich. All participants signed the informed consent. After isolation, PMNs were re-suspended in antibiotic-free cell-culture medium (RPMI-Glutamax, Invitrogen, Life Technologies, Basel, Switzerland) supplemented with 10% heat inactivated human serum at a final density of 2×10^6^ cells/ml. For each experiment, PMNs from a different healthy blood donor were isolated (24–40 years of age, both males and females).

For the experiments *P. gingivalis* was washed once with 1 ml of PBS and opsonized with fresh 30% human serum for 30 min at 37°C, where indicated followed by additional washes with PBS before addition to the PMNs, at multiplicity of infection (MOI = bacteria:PMN ratio) 1, 10 and 100, for 4 h or 18 h. Experiments were carried out in triplicate cultures in V-bottom 96 wells plates and were centrifuged at 380×*g* for 5 min to synchronize adherence of bacteria to PMNs. At least three independent experiments were performed.

### Cytotoxicity Assay

The potential cytotoxicity of PMNs by *P. gingivalis* W50 or its derivative E8 and K1A strains was determined by measuring the extracellularly released lactate dehydrogenase (LDH) activity, over a period of 18 h using the CytoTox 96 Non-radioactive Cytotoxicity Assay (Promega, Mannheim, Germany), according to the manufacturer’s instructions. The absorbance was read at 490 nm by a spectophotometric plate reader (Epoch, BioTek, Luzern, Switzerland). The LDH enzyme activity released from damaged cells into the supernatant was expressed as a percentage of total (intracellular+extracellular) LDH activity.

### RNA Extraction and cDNA Synthesis

Upon completion of the experiments, the PMNs were collected by centrifugation, after removal of the culture supernatants (which were stored at −80°C, until further analysis). The PMN pellets were thereafter washed twice in PBS before lysis. Total RNA was extracted from the collected cell lysates using the RNeasy Mini Kit (QIAGEN, Basel, Switzerland), according to the manufacturer’s instructions. RNA concentration was measured by a NanoDrop 1000 spectrophotometer. One microgram of total RNA was then reverse transcribed into single-stranded cDNA by using M-MLV Reverse Transcriptase, Oligo(dT)_15_ Primers, and PCR Nucleotide Mix according to the manufacturer’s protocol (Promega, Mannheim, Germany), at 40°C for 60 min, and 70°C for 15 min.

### Quantitative Real-time Polymerase Chain Reaction (qPCR)

TaqMan qPCR was employed for gene expression analysis, in a StepOne Real Time PCR System and software (Applied Biosystems, Life Technologies, Basel, Switzerland). Beta-2 microglobulin (B2M) was used as endogenous control in the samples (house-keeping gene). For the amplification reactions, the Applied Biosystems TaqMan Gene Expression Master Mix and Gene Expression Assay kits were used (assay IDs TREM-1: Hs00218624-m1, B2M: Hs00984230-m1). The standard PCR conditions were 10 min at 95°C, followed 40 cycles at 95°C for 15 sec and 60°C for 1 min. The expression levels of the target transcripts in each sample were calculated by the comparative Ct method (2^−ΔCt^ formula) after normalization to the house-keeping gene.

### Measurement of sTREM-1 and Interleukin (IL)-8 by ELISA

The levels of sTREM-1 and IL-8 in the cell-free culture supernatants were measured by commercially available specific enzyme-linked immunosorbent assay (ELISA) kits (DuoSet, R&D Systems, Abingdon, UK). The absorbance at 450 nm was measured using a microplate reader (Epoch, BioTek, Luzern, Switzerland), with a wavelength correction set at 570 nm to subtract background. A standard curve was generated using a four-parameter logistic curve fit for each set of samples assayed.

### TREM-1 Engagement and Antagonism Experiments

The involvement of TREM-1 in the instigation of pro-inflammatory responses was further evaluated, by measuring the secretion of interleukin (IL)-8, as a representative cytokine. For investigating the engagement of TREM-1 in IL-8 production, PMNs were cultured in 96-well flat-bottom plates, which were pre-coated (4 h at 37°C) with either anti-human TREM-1 antibody (10 µg/ml) or an isotype control (10 µg/ml) (R&D Systems, Abingdon, UK, mouse IgG_1_, clone #193015). The PMNs were plated at a density of 2×10^5^ cells per well and challenged with *P. gingivalis* MOI 100, for 18 h. Conversely, the relative involvement of TREM-1 in IL-8 secretion was investigated by the use of its antagonist LP17 peptide (Pepscan Presto B.V., Lelystad, The Netherlands). By this approach, LP17 was added to the cells at concentration 100 ng/ml, simultaneously to *P. gingivalis* challenge (MOI 100).

### Flow Cytometric Analysis for TREM-1 Detection on the PMN Surface

The PMNs were challenged with ascending *P. gingivalis* MOIs for 4 h and 18 h, as described above. At the end of the experimentation, the cells were washed twice, followed by subsequent staining with monoclonal anti-TREM-1 Alexa Fluor 488 (R&D Systems, Abington, UK) on ice for 30 min. The stained cells were washed, re-suspended and the analysis was performed using a FACSCalibur (Becton Dickinson, Oxford, U.K) flow cytometer and FlowJo Software (Tree Star, Ashland, OR, USA).

### Confocal Laser Scanning Microscopy (CLSM) Analysis of TREM-1 Localization

To investigate the localization of TREM-1, as well as *P. gingivalis*, on the PMNs CLSM was used. The cells were cultured on poly-d-lysine-coated dishes (MatTek Corp., Ashton, MA, USA) and challenged with *P. gingivalis* MOI 100, for 18 h. Upon completion of the experiments, the dishes were washed and stained using anti-TREM-1 Alexa Fluor 488 (R&D Systems, Abington, UK) followed by fixation in 4% paraformaldehyde (PFA) for 10 min at room temperature. Thereafter, the discs were processed by fluorescent *in situ* hybridization (FISH) for the detection of *P. gingivalis*, using simultaneously two oligonucleotide probes (Microsynth, Balgach, Switzerland), one specific for *P. gingivalis* (P-Pging1006 labelled at the 5′-end with Cy3), and one universal bacterial probe (EUB338 labelled at the 5′-end with Cy5). The sequences of these probes as well as the standard hybridization conditions are provided elsewhere [26]. For nuclear staining, the samples were further incubated with 1 mg/ml 4′,6-diamidino-2-phenylindole (DAPI; Sigma–Aldrich, Buchs, Switzerland), to counter-stain DNA. The visualization was performed as described previously [22]. In brief, stained samples were examined using a DM IRB/E inverted microscope (Leica Mikroskopie, Wetzlar, Germany), fitted with a UV laser and an Ar laser (both from Coherent Inc., Santa Clara, CA, USA), a He-Ne laser (Uniphase Vertriebs, Eching/Munich, Germany), and a TCS SP5 computer-operated confocal laser scanning system (Leica Lasertechnik, Heidelberg, Germany). Filters were set to 430–470 nm to detect DAPI, to 500–540 nm for Alexa 488, to 570–630 nm for Cy3, and to 660–710 nm for Cy5. Confocal images were obtained using×63 (numeric aperture 1.30) glycerol immersion objective. Z-series were generated by vertical optical sectioning with the slice thickness set at 1.02 µm. Image acquisition was performed in×8 line average mode. Scans were recombined and processed using Imaris 7.3.0 software (Bitplane, Zürich, Switzerland), without any qualitative changes to the raw images.

### Statistical Analysis

A one-way analysis of variance (ANOVA) was used to analyze the statistical significances of the results, using Bonferroni post hoc test for comparisons between individual groups. The data were considered significant at *P*<0.05.

## Results

### Cytotoxic Effects of *P. gingivalis Strains*

Initially, the potential cytotoxic effects of *P. gingivalis* wild-type W50 strain and its gingipain knock-out mutant K1A and E8 strains on the PMN cells were tested, for up-to 18 h, and at MOI of 100. The percentage of extracellularly released LDH was then measured, indicative of cell lysis. It was found that there were no significant differences between the unchallenged PMNs (mean ± SD, 13.0±0.9%) or challenged with *P. gingivalis* W50 (mean ± SD, 11.7±1.9%), *P. gingivalis* E8 (mean ± SD, 12.4±2.2%), or *P. gingivalis* K1A (mean ± SD, 11.1±0.11%).

### Effects of *P. gingivalis* on TREM-1 Expression and sTREM-1 Release by PMNs

**Figure 1:**
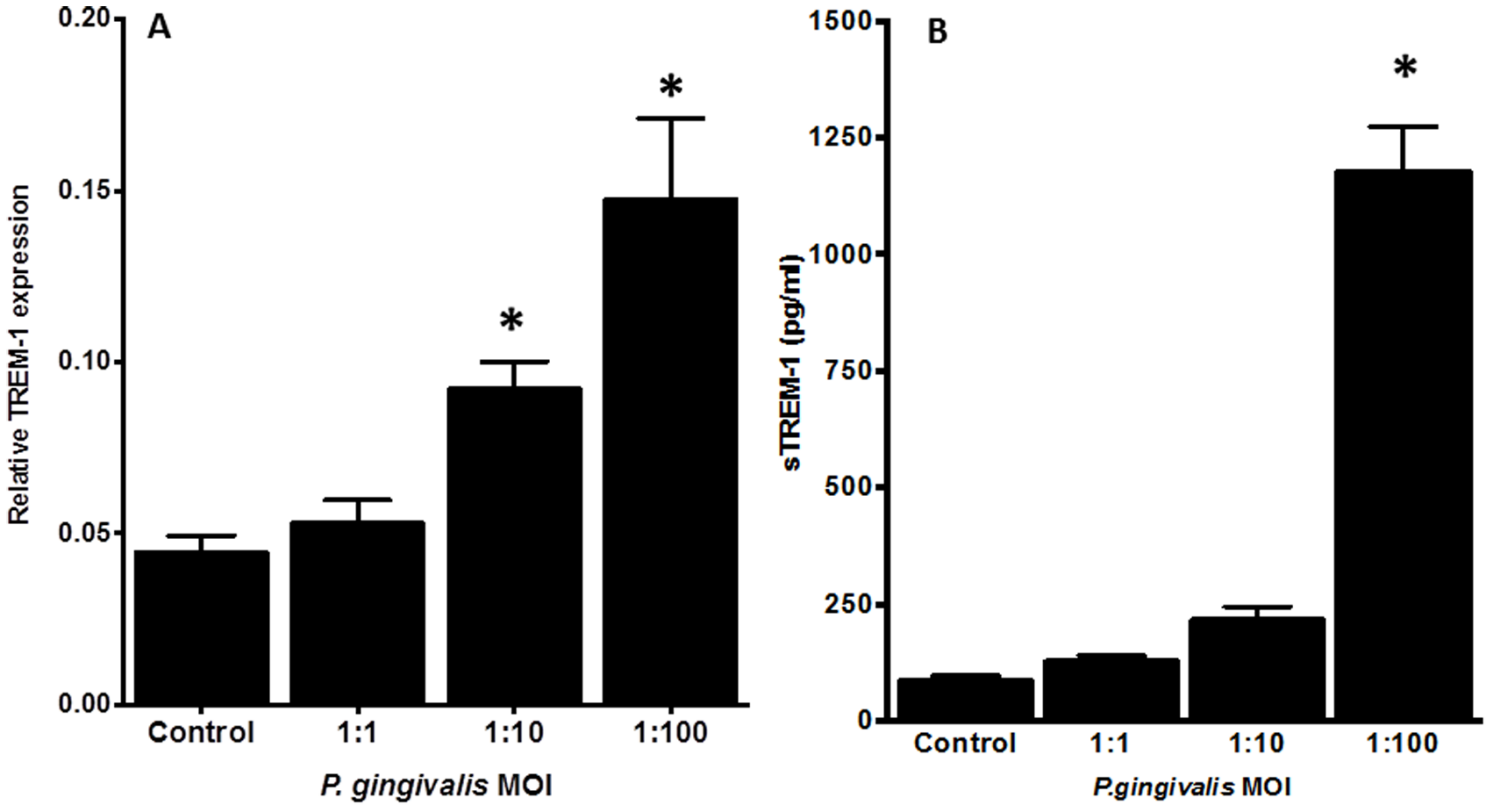
Induction of TREM-1 gene expression and sTREM-1 secretion in PMNs, in response to *P. gingivalis*. PMNs were exposed to *P. gingivalis* W50 at MOI 1, 10 or 100. After 4 h, the gene expression levels of TREM-1 were measured by qPCR analysis, normalized against the expression levels of the house-keeping gene. The results are expressed as the 2^−ΔCT^ formula (A). After 18 h, secretion of sTREM-1 by PMNs into the culture supernatants were measured by ELISA (B). Bars represent mean values ± standard errors of mean (SEM) from three independent experiments. The asterisk represents statistically significant difference between the *P. gingivalis*-challenged and control groups.

The effect of *P. gingivalis* wild-type W50 strain on TREM-1 mRNA expression was investigated. After 4 h, the wild-type *P. gingivalis* W50 strain induced a concentration-dependent up-regulation of TREM-1 expression, which proved to be significant with MOI 10 and MOI 100 (Figure 1A). This was confirmed by the release of sTREM-1 from PMNs following *P. gingivalis* infection. *P. gingivalis* at a MOI 100 caused a significant 13-fold increase of sTREM-1 release, compared to the control, which was absent at lower MOIs (Figure 1B).

**Figure 2:**
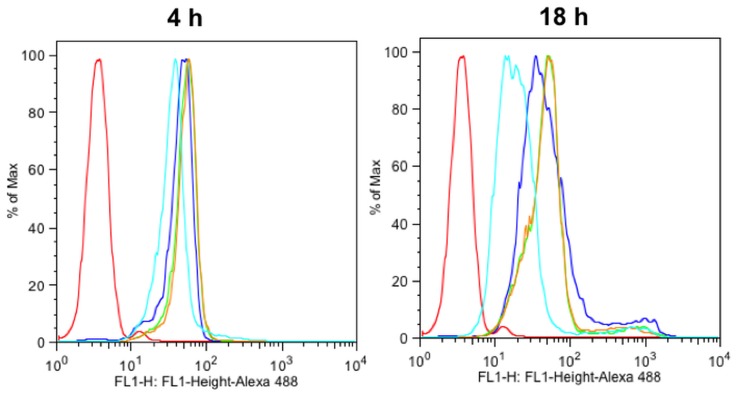
Flow cytometic analysis of PMNs challenged for 4*P. gingivalis* W50 MOI. TREM-1-associated fluorescence on the cell surface was detected by staining with anti-TREM-1-FITC. Results from one experiment are presented (red: unstained cells, dark blue: unchallenged control, green: cells challenged with *P. gingivalis* W50 MOI 1, orange: cells challenged with *P. gingivalis* W50 MOI 10, light blue: cells challenged with *P. gingivalis* W50 MOI 100).

**Figure 3:**
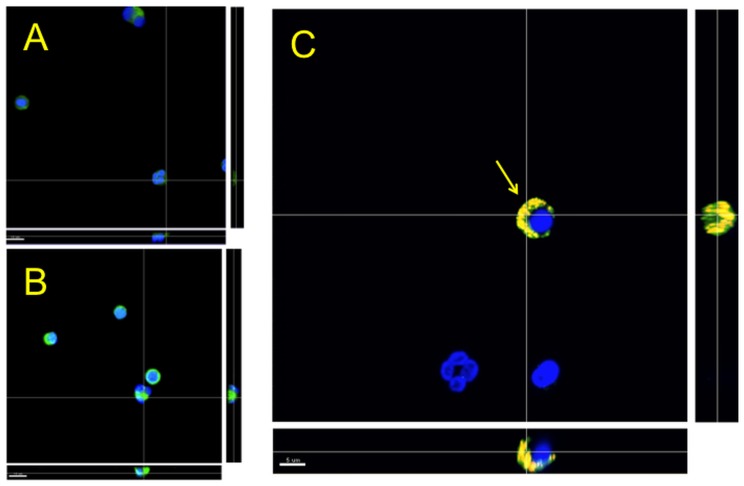
Representative confocal laser scanning microscopy (CLSM) images of PMNs infected with *P. gingivalis* MOI 100 for 18 h. TREM-1 on the cell surface was visualized using anti-TREM-1-FITC (green), and the cell nuclei appear blue due to counter-staining of the DNA with DAPI. Bacteria were stained by FISH with P-Pging1006-Cy3 and universal bacterial probe EUB338-Cy5. Control cells, only DAPI stained (green appears due to autofluorescence) (A), Control cells, stained by both DAPI and anti-TREM-1-FITC (B); Cells infected with *P. gingivalis* W50 (yellow) indicated by arrow, stained by both DAPI and anti-TREM-1-FITC (C). Scales = 10 µm (A, B) and 5 µm (C).

To determine if *P. gingivalis* infection could elicit TREM-1 shedding from PMNs, its cell-surface levels were measured by flow cytometry. Although there were no differences between groups after 4 h, membrane-associated TREM-1 was down-regulated after 18 h in response to increasing MOI of *P. gingivalis* (Figure 2). TREM-1 expression on the PMNs cell surface in response to *P. gingivalis* was further investigated by CLSM. Unchallenged PMNs expressed TREM-1 on their surface as seen by the presence of a strong positive green staining (Figure 3B). However, when the PMNs were infected with *P. gingivalis* MOI 100, the localization of TREM-1 on the cell surface was decreased, and the nuclei appeared more rounded with vivid DAPI staining (Figure 3C). Further, co-staining by FISH with a species–specific, or a generic eubacterial probe, indicated that *P. gingivalis* was localized on the PMNs (Figure 3C, arrow).

### TREM-1 Engagement and Antagonism in *P. gingivalis*-induced IL-8 Production

**Figure 4:**
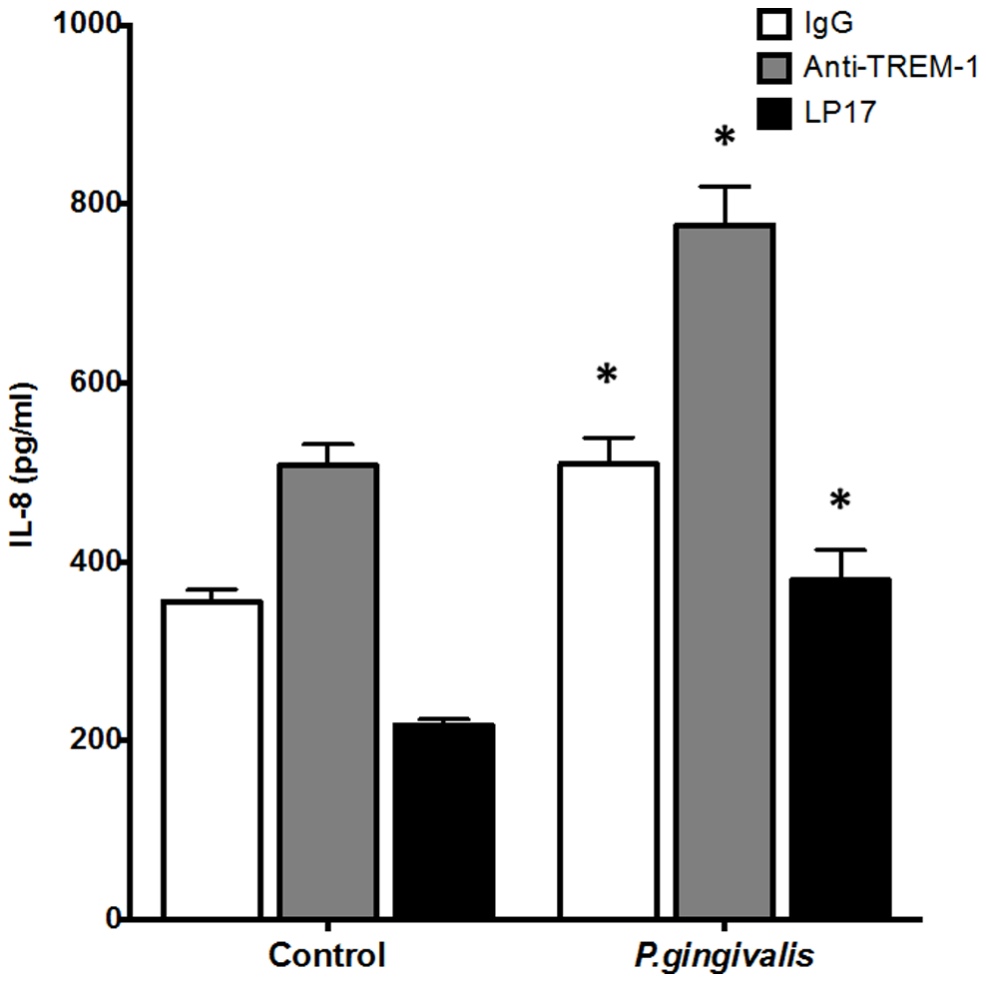
Engagement and antagonism of TREM-1 in IL-8 stimulating-responses by *P. gingivalis*. PMNs were cultured in 96-well plates pre-coated with anti-human TREM-1, or matching IgG_1_ isotype control, or in the presence of LP17 (100 ng/ml) and exposed to *P. gingivalis* MOI 100 for 18 h. Upon completion of the experiments, the cell-free culture supernatants were collected and the concentrations of IL-8 were measured by ELISA. Bars represent mean values ± standard errors of mean (SEM) from three independent experiments. The asterisk represents statistically significant difference between the control and the corresponding *P. gingivalis*-challenged groups.

In order to assess the consequences of TREM-1 engagement on *P. gingivalis*-induced IL-8 release by PMNs the natural ligand of the TREM-1 receptor anti-TREM-1 antibody was used. After 18 h, *P. gingivalis* induced IL-8 secretion by the PMNs which was further enhanced by 35% in the presence of anti-TREM-1 (Figure 4). The presence of anti-TREM-1 alone was adequate to cause a significant enhancement of IL-8 secretion by 30% in absence of *P. gingivalis* compared to the IgG isotype control.

Using a complementary approach, we investigated whether the induction of IL-8 secretion by *P. gingivalis* could be blocked by adding the synthetic peptide LP17 that mimics a highly conserved domain of sTREM-1. We found that the addition of the LP17 antagonist (100 ng/ml) of TREM-1 resulted in 25% reduction of IL-8 secretion by the *P. gingivalis*-challenged PMNs, (Figure 4). The presence of LP17 reduced IL-8 secretion by the cells by almost 30%, even in the absence of *P. gingivalis*.

### Investigation of Gingipain Involvement on TREM-1 Regulation by *P. gingivalis*

**Figure 5:**
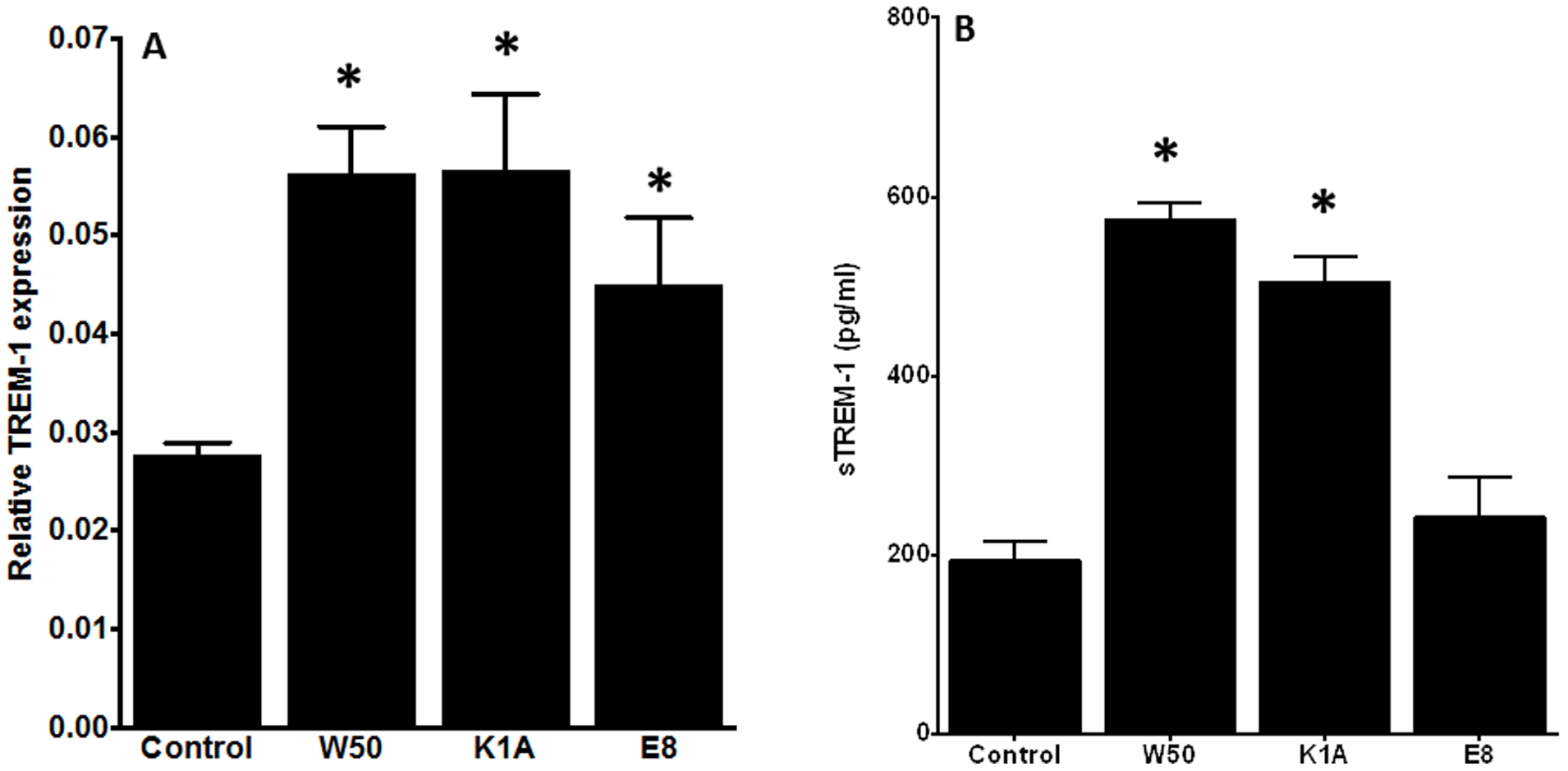
Involvement of gingipains in TREM-1 gene expression and secretion. PMNs were exposed to *P. gingivalis* W50, the Arg-gingipain mutant E8 or the Lys-gingipain mutant K1A strain (MOI 100). After 4 h, the gene expression levels of TREM-1 were measured by qPCR analysis, normalized against the expression levels of the house-keeping gene. The results are expressed as the 2^−ΔCT^ formula (A). After 18 h, secretion of sTREM-1 by PMNs into the culture supernatants was measured by ELISA (B). Bars represent mean values ± standard errors of mean (SEM) from three independent experiments. The asterisk represents statistically significant difference between the *P. gingivalis*-challenged and control groups.

Next, the potential involvement of *P. gingivalis* gingipains in the regulation of TREM-1 was investigated, by employing the Lys-gingipain mutant strain K1A, and the Arg-gingipain mutant strain E8. It was found that both *P. gingivalis* gingipain-mutant strains induced TREM-1 expression in PMNs to a similar extent as their parental wild-type W50 strain, after 4 h of infection (Figure 5A). On the contrary, differences between the different *P. gingivalis* strains were observed on the sTREM-1 secretion levels. Both the *P. gingivalis* wild-type W50 and K1A strains caused increased sTREM-1 release, whereas, in contrast, the E8 strain did not significantly affect sTREM-1 secretion, compared to the control (Figure 5B).

**Figure 6:**
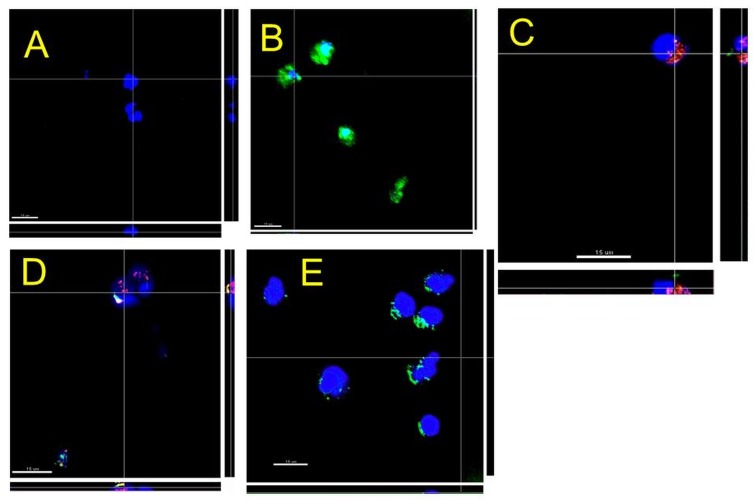
Representative confocal laser scanning microscopy (CLSM) images of PMN cells infected with *P. gingivalis* W50 wild-type, or the Arg-gingipain E8 and Lys-gingipain K1A mutant strains. TREM-1 on the cell surface was visualized using anti-TREM-1-FITC (green), and the cell nuclei appear blue due to counter-staining of the DNA with DAPI. Bacteria were stained by FISH with P-Pging1006-2-Cy3 probe and Cy5-labelled universal bacterial probe EUB338. Control cells, only DAPI stained (A); control cells, stained by both DAPI and anti-TREM-1-FITC (B); cells infected with *P. gingivalis* W50 (red) (C); cells infected with *P. gingivalis* K1A (red) (D); cells infected with *P. gingivalis* E8 (E) (Scales = 15 µm).

The localization of PMN cell-bound TREM-1 in response to the *P. gingivalis* gingipain mutant strains was further confirmed by CLSM (Figure 6). In the unchallenged control cultures, there was a strong positive green staining for TREM-1, associated with the surface of the PMNs (Figure 6B). However, when the cells were infected with *P. gingivalis* wild-type W50 or the Lys-gingipain mutant K1A strain, TREM-1 was no longer detected on the cell surface (Figure 6C–D). In contrast, in PMNs challenged with the *P. gingivalis* Arg-gingipain mutant strain E8, partial TREM-1 staining was detected on the cells (Figure 6E). This is complementary to the finding by ELISA, showing decreased sTREM-1 release in response to the E8 strain. Collectively, these data may indicate that absence of the Arg-gingipain from *P. gingivalis* may prevent shedding of TREM-1 from the PMN cell surface into the culture supernatant.

### Effect of *P. gingivalis* Gingipains on sTREM-1 Degradation

**Figure 7:**
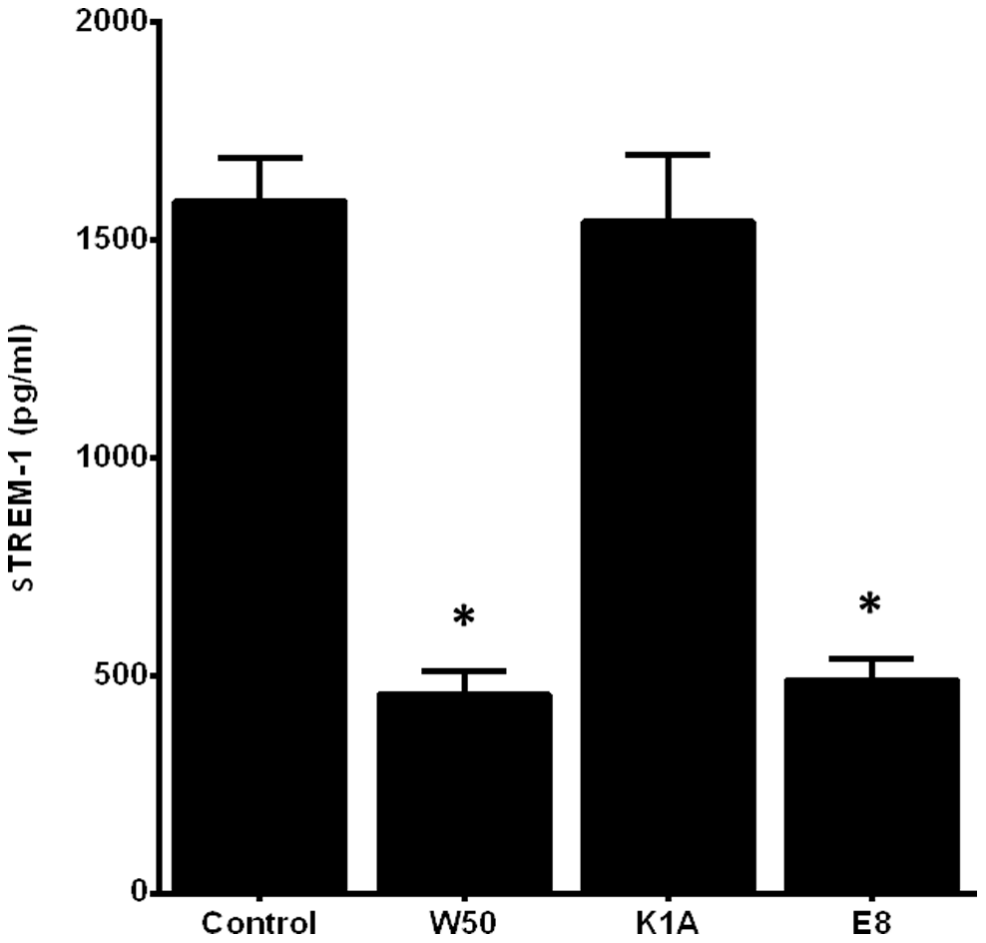
Effect of *P. gingivalis* gingipains on sTREM-1 degradation. Recombinant human sTREM-1 (2000 pg/ml) was spiked into cell culture media in absence or presence of *P. gingivalis* W50 wild-type, Arg-gingipain E8 or Lys-gingipain K1A mutant strain (MOI 100) for 18 h. Bars represent mean values ± standard errors of mean (SEM) from one experiment in triplicate. The asterisk represents statistically significant difference between the control and the *P. gingivalis*-challenged groups.

The capacity of *P. gingivalis* to degrade sTREM-1 was further tested. A defined concentration of recombinant sTREM-1 (2000 pg/ml) was spiked into cell culture media in the absence or presence of any of the three *P. gingivalis* strains, over a period of 18 h. Significant reduction of sTREM-1 concentrations occurred in the presence of *P. gingivalis* wild-type and Arg-gingipain mutant E8 strain, but not in the case of the Lys-gingipain mutant K1A strain (Figure 7). The resulting sTREM-1 concentration accounted for approximately 30% of control levels. These findings are suggestive of the capacity of Lys-gingipain, but not Arg-gingipain, to degrade sTREM-1.

## Discussion

We found that *P. gingivalis* regulates TREM-1 in PMNs, with potential implications in down-stream inflammatory responses. *P. gingivalis* induced TREM-1 gene expression independent of its gingipains, followed by sTREM-1 release from the cell surface, in a gingipain-dependent manner. *Porphyromonas gingivalis* is well known for its capacity to modulate innate immunity in periodontal inflammation via PMNs, resulting in survival benefits [14]. In agreement with earlier reports *in vivo* [27] *P. gingivalis* did not cause any cytotoxic effects. There is also evidence that *P. gingivalis* can decrease [28], delay [29]–[31] and inhibit [32] PMN apoptosis, in addition to preventing the clearance of apoptotic cells [27], [33]–[35], and that activation of TREM-1 can exert a protective effect against apoptosis [36]. Although these findings support an anti-apoptotic effect of *P. gingivalis*-induced TREM-1 activation, a pro-apoptotic effect cannot be excluded, due to the long period of PMN exposure, also applied in other studies [37], [38], and the rounding of the PMN cell nuclei, particularly in presence of *P. gingivalis*, as viewed by CLSM. *P. gingivalis* induced TREM-1 gene expression independent of its gingipains, but also sTREM-1 release from the cell surface into the culture medium, in a gingipain-dependent manner. These findings are in agreement with previous reports demonstrating that PMNs are involved in sTREM-1 generation during other infections [5], [39]. Moreover, recent findings demonstrate an increased sTREM-1 release by monocytic cells, in response to *P. gingivalis* [22], [40]. The occurrence of sTREM-1 in biological fluids appears to be an important predictor and diagnostic marker for sepsis, bacterial and fungal pneumonia [41]. A positive association has been shown between periodontitis and the levels of sTREM-1 in serum and saliva, potentially revealing a link between systemic and oral inflammation [10]. Interestingly, a positive correlation is shown between gingival crevicular fluid site-specific levels of sTREM-1 and the levels of *P. gingivalis* in subgingival biofilms [42]. The findings of the present *in vitro* study on PMNs reveal a potential mechanistic pathway between *P. gingivalis* and the increased levels of sTREM-1.

It was further confirmed, by engagement and antagonism experiments, that TREM-1 propagated pro-inflammatory cytokine production, representatively demonstrated by IL-8 in the present study. Earlier studies have also demonstrated that, in response to bacterial challenge, TREM-1 engagement enhances the release of IL-8 from PMNs [5], [23], or monocytic cells [8], [22], [40]. Since IL-8 is a potent chemokine, its enhanced secretion mediated by TREM-1 may consequently lead to further PMN recruitment at the affected site (i.e. gingival sulcus or periodontal pocket), in an attempt to tackle with the increasing bacterial challenge.

We identified the gingipains as the responsible *P. gingivalis* virulence factor for the shedding of sTREM-1 by PMNs. Gingipains are considered detrimental in the capacity of *P. gingivalis* to evade host innate immune responses, by contributing to its capacity to resist complement elimination, prevent blood clotting, and acquire essential nutrients [43] and activate matrix metalloproteinases [44]. It was found that the *P.gingivalis* Arg-gingipain mutant strain was unable to enhance sTREM-1 release by the PMNs, marked by a concomitant intense TREM-1 presence on the cells, in contrast to the effects of the wild-type or the Lys-gingipain mutant strain. These results indicate that sTREM-1 shedding from the cell surface is attributed to its Arg-gingipains. Nevertheless, as gingipains are also known to degrade several proteins, we evaluated the capacity of *P. gingivalis* to degrade known concentrations of sTREM-1. We found that sTREM-1 was resistant to degradation by the Lys-gingipain mutant strain, as opposed to the Arg-gingipain mutant, or the wild-type *P. gingivalis* strain. This indicates that the Lys-gingipain is responsible for sTREM-1 degradation, once this molecule is shed from the cell surface by the Arg-gingipain. These findings are collectively in line with earlier studies confirming the enzymatic properties of gingipains in cleaving and degrading complement factors, immunoglobulins. metalloproteinases, the fibronectin-integrin binding, toll-like receptors and other molecules [43], [45]–[49]. Both the Lys-gingipain and the Arg-gingipain are also shown to be able to cleave IL-8 and modulate its biological activity, as well as to degrade and inactivate tumor necrosis factor-α [50], [51].

The capacity of *P. gingivalis* to differentially regulate molecules via its gingipains may favor the evasion of the innate immunity mechanisms that would normally result in its phagocytosis and killing. In the instance of TREM-1 as revealed in the present experimental system, *P. gingivalis* may employ its Arg-gingipain to shed-off sTREM-1 from the PMNs, thus amplifying the inflammatory response. On the other hand degradation of sTREM-1 by the Lys-gingipain may eliminate the ability of PMNs to propagate an inflammatory response or to efficiently accomplish their anti-bacterial actions, including phagocytosis. The net outcome of TREM-1 regulation in PMNs by *P. gingivalis* may depend on the stage of infection. In early stages, via its Arg-gingipain, *P. gingivalis* may cause a sTREM-1-supported innate immune response, creating an inflammation-rich environment for its survival. In later stages, where the excessive inflammatory response may compromise its survival, *P. gingivalis* may employ its Lys-gingipain to control this and remain stealth. Hence, dual regulation of sTREM-1 release and degradation by two different ginigpains may be a novel mechanism by which *P. gingivalis* evades the host defenses and establishes chronic periodontal inflammation.
